# Robotic Rehabilitation and Transcranial Direct Current Stimulation in Children With Bilateral Cerebral Palsy

**DOI:** 10.3389/fresc.2022.843767

**Published:** 2022-02-25

**Authors:** Liliane Raess, Rachel L. Hawe, Megan Metzler, Ephrem Zewdie, Elizabeth Condliffe, Sean P. Dukelow, Adam Kirton

**Affiliations:** ^1^University Children's Hospital Zurich, Zurich, Switzerland; ^2^Clinical Neurosciences, Alberta Children's Hospital, Calgary, AB, Canada; ^3^Department of Clinical Neurosciences, Hotchkiss Brain Institute, University of Calgary, Calgary, AB, Canada; ^4^Alberta Children's Hospital Research Institute, Calgary, AB, Canada; ^5^Calgary Pediatric Stroke Program, University of Calgary, Calgary, AB, Canada; ^6^Department of Pediatrics, Cumming School of Medicine, University of Calgary, Calgary, AB, Canada

**Keywords:** children, bilateral cerebral palsy, robotic rehabilitation, transcranial direct current stimulation, pediatric

## Abstract

**Aim:**

To identify challenges of combining robotic upper extremity rehabilitation with tDCS in children with upper extremity bilateral cerebral palsy (CP) by assessing feasibility, tolerability and safety.

**Methods:**

This was an unblinded, open-label, pilot clinical trial. Participants completed 10 × 1 h sessions of robotic rehabilitation combined with motor cortex anodal tDCS. Feasibility, acceptability and practicality, were assessed including the number of participants completing the protocol, factors limiting participation, time required for sessions, and completion of functional assessments and tolerability scales. To assess safety, standardized clinical and robotic measures of sensorimotor function were performed. The trial was registered at clinicaltrials.gov (NCT04233710).

**Results:**

Eight children were recruited (mean age 8y ± 1.8y, range 6–11 years) and 5 completed the intervention. There were no serious adverse events. One child developed focal seizures 6 weeks after the trial that were deemed to be unrelated. Barriers to completion included time and scheduling demands and patient factors, specifically cognitive/behavioral impairments and dyskinesia. No decline in clinical function was appreciated.

**Conclusions:**

Robotic upper extremity rehabilitation combined with tDCS may be feasible in children with bilateral CP. Careful participant selection, family engagement, and protocol adaptations are recommended to better understand the feasibility and tolerability of future trials.

## Introduction

Cerebral palsy (CP) is the leading form of lifelong motor disability and affects millions of people worldwide. Bilateral spastic CP is the most common pattern (1). Moderate to severe impairment of upper extremity function [Manual Ability Classification System (MACS) III-V] occurs in up to half of all children with bilateral spastic CP (2). Rehabilitation aims to restore meaningful function and maximize participation but current options are limited (3).

Progress is being made toward novel, evidence-based upper extremity therapies (4). Most of the studies investigated these new therapies in children with unilateral CP but whether these tools translate to treating children with bilateral impairments has not been well-studied (3). Bilateral CP brings additional challenges due to a higher burden of comorbidities in this patient group, such as cognitive and vision impairment (5). The inclusion of children with bilateral CP in trials for novel upper extremity studies is essential to ensure equitable access to remedial therapies for an under-represented group with disproportionate functional impacts of injury.

Robotic therapy tasks may be able to train performance by using intensive task-specific training, targeting specific impairments, grading difficulty levels and tracking improvements (6, 7). The Kinarm exoskeleton (Kingston, Canada) was developed to quantify sensorimotor function in individuals with neurologic impairments and has also been used successfully as a training tool in a pilot study in adults with stroke (8).

Non-invasive brain stimulation can modulate neuroplasticity and motor learning in both adults and children (9). Transcranial direct current stimulation (tDCS) is a leading approach given its relative simplicity and strong safety profile (10). Current evidence suggests that tDCS is a modulator of natural, endogenous plasticity which must be invoked through simultaneous motor training (11). The application of tDCS over the contralateral motor cortex has been shown to enhance motor learning in healthy adults and children (12). Preliminary evidence for tDCS in bilateral CP suggests possible improvements of gait and mobility (13). Two small trials have investigated the application of tDCS for upper extremity function in children with bilateral CP but neither paired tDCS with active motor learning interventions (14, 15). The combination of tDCS with robotic training has not been previously reported in children. Accordingly, we completed a pilot clinical trial to evaluate the feasibility (16), tolerability, and safety of combining tDCS with robotic therapy in school-aged children with bilateral CP.

## Materials and Methods

**Figure 1:**
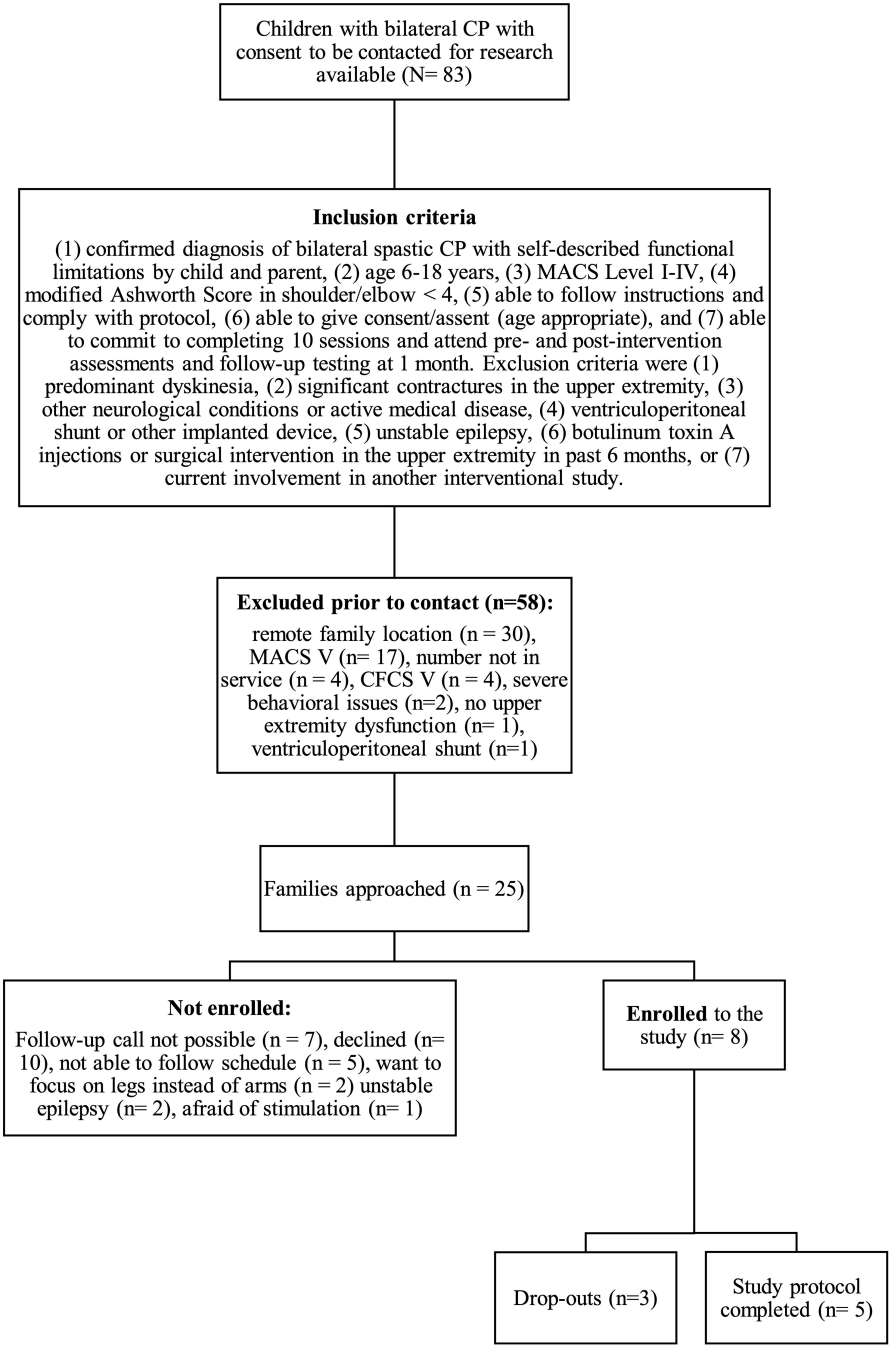
Study recruitment process. CP, cerebral palsy; MACS, Manual Ability Classification System; CFCS, Communication Function Classification System.

We conducted a non-blinded, one-arm interventional trial. Participants were recruited from outpatient clinics at the Alberta Children's Hospital between October 2019 and February 2020. Diagnosis of bilateral CP was confirmed and classified by reviewing medical records and clinically obtained MRI. For inclusion criteria see Figure 1. Parent(s) or guardian(s) were approached via phone initially and recruited with written informed consent. Participants attended for 10 consecutive weekdays for a 60-min session of robotic rehabilitation combined with tDCS, in addition to three assessment visits (pre-assessment, post-assessment and follow- up after 1 month).

Upper extremity training was performed with the Kinarm Exoskeleton robot (Kinarm, Kingston, Canada). The Kinarm exoskeleton supports the weight of the limb through forearm and upper arm supports. The exoskeleton allows free, gravity-eliminated movement in the horizontal plane. Representations of the hand and custom tasks were projected on a horizontally-oriented visual display that is approximately at the level of the participant's sternal notch when seated in the robot. Eight different training tasks were used (see Supplementary Material). Anodal 1x1 tDCS was applied for the first 20 min of each session. The child and caregivers identified the target limb for training, recognizing that patient-centered goals would differ in laterality in children with bilateral CP. The motor cortex contralateral to the chosen arm was then targeted. Current was delivered with a Soterix DC stimulator (Soterix, NYC) via 2 saline-soaked sponge electrodes with the anode placed over M1 and the cathode over the contralateral forehead, both held in place by a custom-sized headstrap. M1 location was approximated using the 10/20 EEG system to map targets of left (C3) or right (C4) (17). Current was ramped up for 45 s from 0 to 1 mA. Stimulation remained on for 20 min followed by a 45 s ramp-down.

Feasibility in terms of acceptability and practicality was assessed by the following measures: the number of participants who completed the full study protocol, enrollment and dropout rates and reasons for dropout, a pediatric brain stimulation tolerability questionnaire administered after each session, by which the child ranked their tDCS session against 8 common childhood experiences (9). To evaluate practicality, we measured the time required for set up and training 6 and documented whether assessments were completed successfully or not.

The following assessments of arm function were conducted at baseline (<14 days prior to the start of intervention) and twice after the intervention: 1 week (range 1–10 days) and 1 month (within 7 days) after the intervention:

- Melbourne Assessment 2 (18). Hand function was excluded from the assessment (excluded Items 3, 4, 8), because training did not target the hand function. Maximum scores of the subscores *Range of Movement, Accuracy* and *Fluency* were adjusted accordingly.
- Box and Block Test (19). The test was performed in a single trial for each arm beginning with the less affected side.
- ABILHAND Kids Questionnaire (20). Parents completed the questionnaire at pre-, post, and follow-up assessments.
- Kinarm assessment task (21). The standardized robotic visually guided reaching task was administered on each arm and 4 different parameters were analyzed: Initial direction angle, speed maxima count, path length ratio, and maximal speed.

Assessments were regarded as completed when measures were available from each baseline, 1 week and 1 month follow-up. Descriptive statistics were used. Analysis was performed using R statistical software (R Studio Version 1.2.5001).

## Results

**Figure 2:**
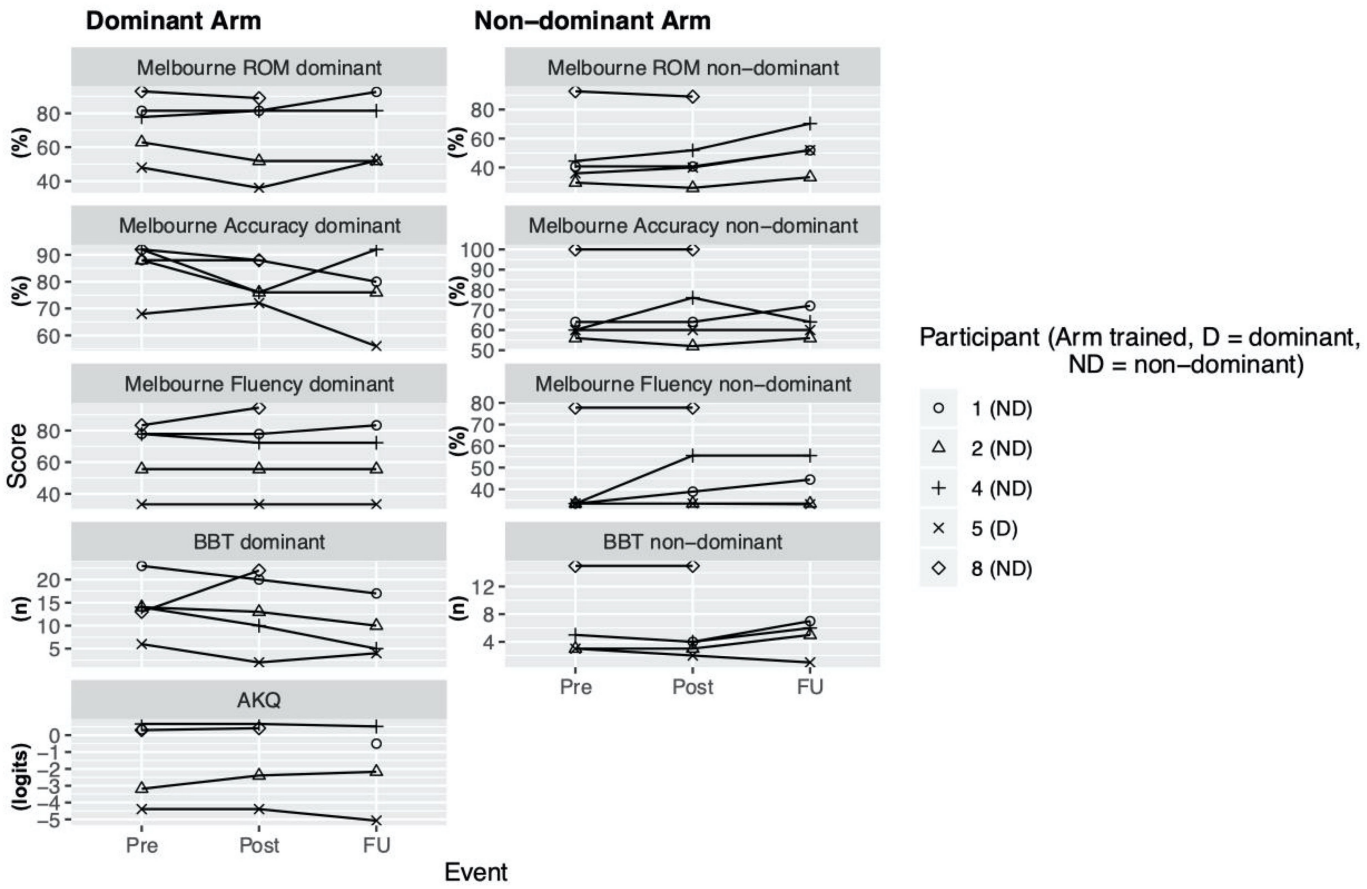
Clinical assessment scores. Scores at assessment events Pre (= before the intervention), Post (= 1 week after intervention), delayed follow up (FU) (= 1 month after intervention) for Melbourne Assessment 2 (Subscores: *Range of Movement (ROM), Accuracy, Fluency*), Box and Block test (BBT), ABILHAND Kids Questionnaire (AKQ).

**Figure 3:**
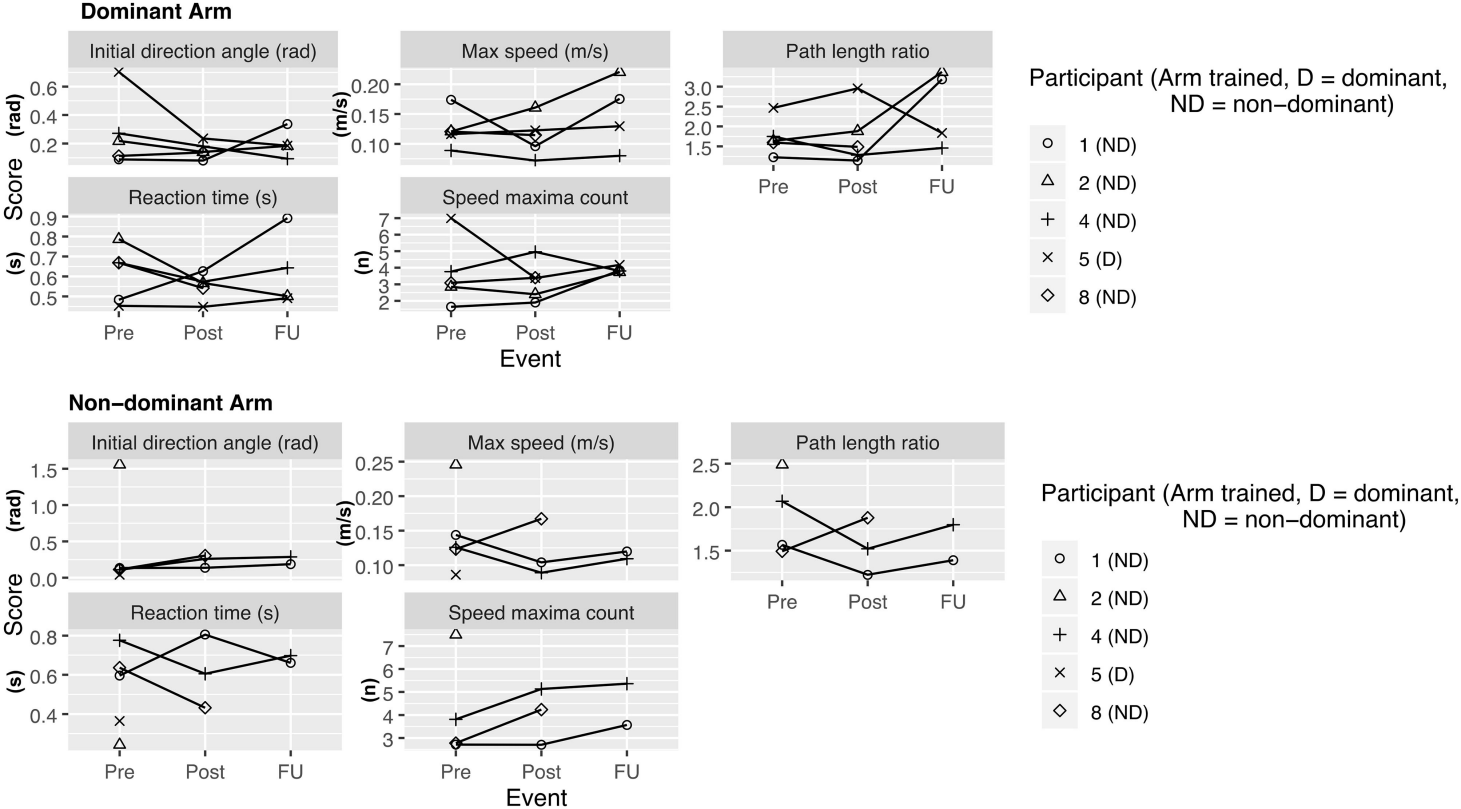
Kinarm assessment—visually guided reaching task. Scores at assessment events Pre (= before the intervention), Post (= 1 week after intervention), delayed follow up (FU) (= 1 month after intervention). Lower values at retest indicate improvement for all variables except for maximum speed, for which an increase is indicative of improvement.

During the enrollment period (October 2019–February 2020), 8 families consented (see Figure 1). Characteristics of the study group are summarized in Table 1. Five children completed the entire study protocol. Participant 8 missed the final follow- up assessment due to the coronavirus pandemic; this participant was considered as complete in terms of feasibility because neither the study protocol nor the child's incapability led to the cancellation. Reasons for not completing the intervention were (a) decrease in energy and appetite (Participant 3), (b) dyskinetic movement disorder combined with insufficient cognitive abilities for cooperation (Participant 6) and (c) insufficient cognitive abilities (Participant 7). Participant 3 dropped out after 3 sessions due to a decrease in energy and appetite and need for extra sleep. The participant was reviewed by the study PI and referred to their pediatrician for an assessment the following week. By that point, he had returned to his normal baseline and an intercurrent illness was suspected. The same child then presented to care 6 weeks later with focal seizures with impaired awareness. He was immediately assessed by a pediatric epileptologist who was informed by both the parent and the study PI about the trial details and was provided with a copy of the protocol. Their independent assessment concluded that the child may have had focal epilepsy prior to trial involvement and, even if not, the new onset of remote symptomatic epilepsy in a child with such a neurological history was considered common and well-explained by their known underlying injury. The event was therefore deemed to be unrelated to the trial. No serious adverse events occurred. Forty-eight training sessions were completed by the five participants who completed the study protocol. The mean total visit time was 60 ± 10 min (range 40–85). The mean set up time was 8 ± 4 minutes (range 2–25) for the Kinarm exoskeleton and 4±4 minutes (range 1–20) for the tDCS headset. The time devoted to training ranged between 22 and 60 min with a mean of 38 ± 8 min. Reasons for shortened sessions included need for breaks, prolonged set up time or early tiredness of participants. In 20 of 51 sessions, breaks were needed with break time varying between 1 and 15 min. The mean training time decreased over the course of the study with 44 min in session 2 and 32 min in session 10. Average tolerability ranking fell between a birthday party and watching TV. Itching of the scalp was reported in all individuals at least once and at some point in 45% of sessions. Duration of itching was <2 min in 45%, >2 min in 45% and 20 min (total stimulation time) in 9%. Tingling of the scalp was reported in 1 participant. The ABILHAND Kids Questionnaire was completed only in 4 of 5 participants, with one parent forgetting to bring back the assessment form. The Melbourne Assessment was completed in all participants and the Box and Block Test in 4 of 5 participants with 1 participant refusing to do the Box and Block Test with the non-dominant arm at baseline, because the task was perceived as too difficult. The robot assessment task was completed in 3 of 5 children, with missing measures of 2 participants because the task was too difficult for their non-dominant arm, even with multiple trials allowed. A qualitative evaluation of these mixed measures suggested no consistent decreases in sensorimotor function (see Figures 2, 3 for individual assessment scores).

**Table 1:** Participant demographics and baseline characteristics.

	**Participant 1**	**Participant 2**	**Participant 3**	**Participant 4**	**Participant 5**	**Participant 6**	**Participant 7**	**Participant 8**
Gender	Male	Male	Male	Male	Female	Female	Male	Female
Age (years)	8y 11m	6y 10m	8y 1m	6y	10y 4m	9y 7m	11y 2m	7y 1m
CP subtype	Spastic	Spastic	Spastic	Spastic	Spastic	Spastic-dyskinetic	Ataxic	Spastic
GMFCS level	III	IV	II	IV	IV- V	IV	II	II
MACS Level	II	III	II	II	IV	IV	III	II
Selected arm for training	ND	ND	ND	ND	D	D	D	ND
CFCS level	I	III	III	II	II	IV	IV	II
Education	Attends conventional school and classroom with no learning concerns	No assessment of learning abilities/disabilities	Attends conventional school and classroom with a support worker for learning impairments	No assessment of learning abilities/disabilities	Attends conventional school and classroom with a support worker for learning impairments	Attends conventional school in a modified classroom for support of learning impairments	Attends conventional school and classroom with a support worker for learning impairments	Attends conventional school and classroom with a support worker for learning impairments
Comorbidities and impairments	Epilepsy	Strabismus	Epilepsy, strabismus	Epilepsy, strabismus	None	Epilepsy	Epilepsy, Angelman Syndrome	Epilepsy,
Medication	Antiepileptic	Antispastic	None	Antispastic	Antispastic	Antiepileptic	Antiepileptic and neuro-psychotropic	None
Clinical MRI classification	White matter injury of prematurity	White matter injury of prematurity	None	HIE: deep gray and watershed lesions	None	HIE: deep gray and watershed lesions	White matter injury of prematurity	HIE: deep gray and watershed lesions

*CP, cerebral palsy; GMFCS, Gross Motor Function Classification System; MACS, Manual Ability Classification System; CFCS, Communication Function Classification System; D, dominant arm; ND, non-dominant arm; HIE, hypoxic-ischemic encephalopathy*.

## Discussion

We conducted a non-blinded one-arm interventional trial of combined robotic upper extremity therapy and tDCS in children with bilateral CP. The study protocol was feasible for five participants. However, recruitment rates were modest and 3 participants were unable to complete all sessions, all of which are compelling reasons to critically review the study design, participant selection, and the selected assessments.

### Participant Selection

Severe cognitive and communication impairment (CFCS IV) and dyskinetic movement disorder limited participation for 2 participants. This finding highlights the heterogeneity in functional level among children with bilateral CP. Tailoring therapy to the individual creates challenges for clinical trials. This pilot study shows that children with spastic CP and severe motor impairment (MACS IV) and mild cognitive and communication impairment might be the patient group to focus on in future similar trials.

### Acceptability of the Study Schedule

The intensive 10-weekday schedule led to 5 families declining to consent and participate. Based on verbal feedback from families, adaption of the study schedule (e.g., 1–2 sessions per week) is recommended to allow participation for more families. However, this drives away from a more intense intervention which probably leads to better outcomes. Training time decreased over the course of the intervention, mainly because the children were bored with repetition of the same games every day. This further supports the suggestion to stretch the study schedule out to a couple of weeks with less sessions per week, but also suggests that a range of content is needed to engage children of varying developmental levels. Tolerability scores of the sessions, especially of the tDCS, were comparable to previous reports (9, 10).

### Practicality of the Study Schedule

Visit times averaged about 1 h per day with modest setup times is reasonable, especially in our particularly demanding patient profile (young children, severely impaired). The time devoted to training demonstrated a large range, which might also affect outcomes in future trials, as not all the children received the same amount of training. The Kinarm appears well-suited to be combined with tDCS as the patient remains seated during the training within the room, facilitating simultaneous application of tDCS during training. The Kinarm exoskeleton was used for motor training with even severely impaired children up to MACS Level IV and GMFCS Level IV-V.

The ABILHAND Kids Questionnaire and Melbourne Assessment are suggested as the assessments with the strongest evidence of validity and reliability when assessing upper extremity function in children with bilateral CP (22). In our study these two assessments were found to have the best rate of successful completion. The Box and Block Test and Kinarm visually-guided reaching task turned out to be difficult for more severely impaired children, respectively, especially for their non-dominant arm and thus probably not a suitable assessments.

### Arm Function

As a group, participants did not have an overall decrease in function after intervention of either arm, providing preliminary indications of safety. Due to the small sample size, we are not able to discriminate whether these measures reflect daily variance or actual change scores due to the intervention.

### Limitations

Generalizability is limited by a small sample size and the results of this young participant group cannot be extrapolated to older children.

## Conclusion

We provide preliminary evidence that robotic upper extremity rehabilitation combined with tDCS may be feasible in children with bilateral CP. Careful participant selection, family engagement, and protocol adaptations are recommended to better understand the feasibility and tolerability of future trials.

## Data Availability Statement

The raw data supporting the conclusions of this article will be made available by the authors, without undue reservation.

## Ethics Statement

The studies involving human participants were reviewed and approved by Conjoint Health Research Ethics Board (CHREB), University of Calgary. Written informed consent to participate in this study was provided by the participants' legal guardian/next of kin.

## Author Contributions

LR conducted all patient recruitment, collected data, designed the analysis, analyzed the data, drafted the first draft of the manuscript and edited subsequent drafts, and generated all figures and tables. RH conceptualized the study, teached LR in using the Kinarm robot and how to collect and analyze data of the latter and revised the manuscript critically for important intellectual content. MM conceptualized the study regarding the question which assessments should be used, acquired data and revised the manuscript critically for important intellectual content. EZ teached LR in using the tDCS, contributed to the interpretation of data and revised the manuscript critically for important intellectual content. EC conceptualized the study, manages the research cohort and revised the manuscript critically for important intellectual content. SD conceptualized the study and revised the manuscript critically for important intellectual content. AK conceptualized the study, oversaw data collection, contributed to the design of analysis, and edited the manuscript. All authors have approved the manuscript for submission and agree to be accountable for all aspects of the work.

## Funding

This study was part of a research elective of LR, that was funded by the Swiss Foundation for Children with Cerebral Palsy (Schweizerische Stiftung für das cerebral gelähmte Kind) and the Alumni-Organization of the Faculty of Medicine, University of Zurich. RH was supported by a Thrasher Research Fund Early Career Award.

## Conflict of Interest

SD has received compensation as a consultant for Promethus Medical and Sinntaxis. He has received compensation from Ipsen for consultation related to spasticity. Further he receives operating grants from the Heart and Stroke Foundation, Canadian Institutes of Health Research and the University of Calgary. The remaining authors declare that the research was conducted in the absence of any commercial or financial relationships that could be construed as a potential conflict of interest.

## Publisher's Note

All claims expressed in this article are solely those of the authors and do not necessarily represent those of their affiliated organizations, or those of the publisher, the editors and the reviewers. Any product that may be evaluated in this article, or claim that may be made by its manufacturer, is not guaranteed or endorsed by the publisher.
