# Supplementary material for: Robotic Rehabilitation and Transcranial Direct Current Stimulation in Children With Bilateral Cerebral Palsy
Source: Front Rehabil Sci. 2022 Feb 25;3:843767. doi: 10.3389/fresc.2022.843767 (PMC9397997; doi:10.3389/fresc.2022.843767)
Supplement: Supplementary file 1 [file Data_Sheet_1.docx]

**KINARM custom training tasks**

Task 1: Visually guided reaching with assistance or resistance: Trains ability to make goal-directed movements to targets. The robot assists the subject to targets if they have difficulty and resists movement when they become more proficient.

Task 2: Virtual Soccer: Trains bimanual motor function. Balls arise from the bottom of the display and the subject “kicks” the balls into a goal using a virtual hand paddle. The size of the balls, goal, and paddle, as well as ball speed can be altered to change difficulty.

Task 3: Shape Tracking: Trains ability to generate arbitrary spatial motions. Subjects move their hand to follow a target moving around circles, ellipses, etc. Movement speed and shapes are modified to increase the difficulty.

Task 4: Whack-a-mole: Mole-like objects arise on the screen and swell. Subjects hit them with a virtual paddle. Moles burst when they are hit or reach a given size. Size, placement, and number of moles are modified to increase task difficulty.

Task 5: Table Tennis: Trains rapid bimanual visuomotor skills. Paddles in the sagittal plane are attached to each hand and subjects must hit a ball back and forth between the two hands. Ball speed increases and paddle size decreases with time.

Task 6: Balancing Act: Trains bimanual motor skills. Subjects balance a moving ball on a virtual bar connected to each hand. Subjects must move the ball to spatial locations in the environment. Increases in ball ‘gravity’ make the task harder.

Task 7: Proprioceptive reaching: Trains the ability to make-goal directed movements in specific directions. The robot applies small forces when a subject reaches with an inappropriate trajectory to gently correct the movement back to an optimal trajectory. The forces applied serve as proprioceptive reminders that the subject has gone off course. Corrective loads and target positions can be modified to increase task difficulty.

Task 8: Hand Ball: Trains unimanual coordination. One hand has a paddle and bounces a ball off a virtual wall. Ball size, speed, and paddle size can be modified to adjust task difficulty.
